# Supplementary material for: Multiscale structural and electronic control of molybdenum disulfide foam for highly efficient hydrogen production
Source: Nat Commun. 2017 Apr 12;8:14430. doi: 10.1038/ncomms14430 (PMC5394285; doi:10.1038/ncomms14430)
Supplement: Supplementary Information — Supplementary Figures, Supplementary Tables and Supplementary Methods [file ncomms14430-s1.pdf]

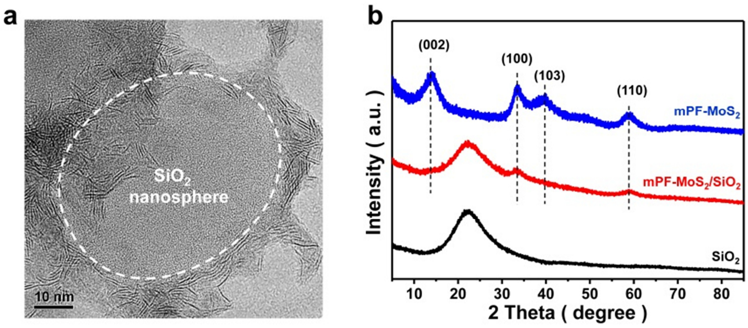

Supplementary Figure 1. (a) HRTEM image of mPF-MoS_2/SiO_2 with inset showing the profile of SiO_2 nanosphere by dashed line. (b) XRD patterns of mPF-MoS_2, mPF-MoS_2/SiO_2 and SiO_2, respectively.

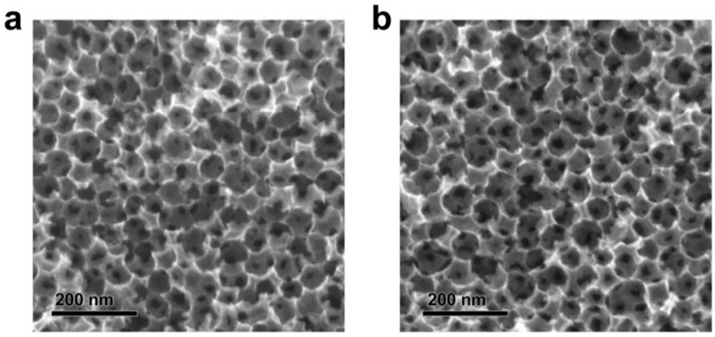

Supplementary Figure 2. (a, b) SEM images of mPF-MoS_2.

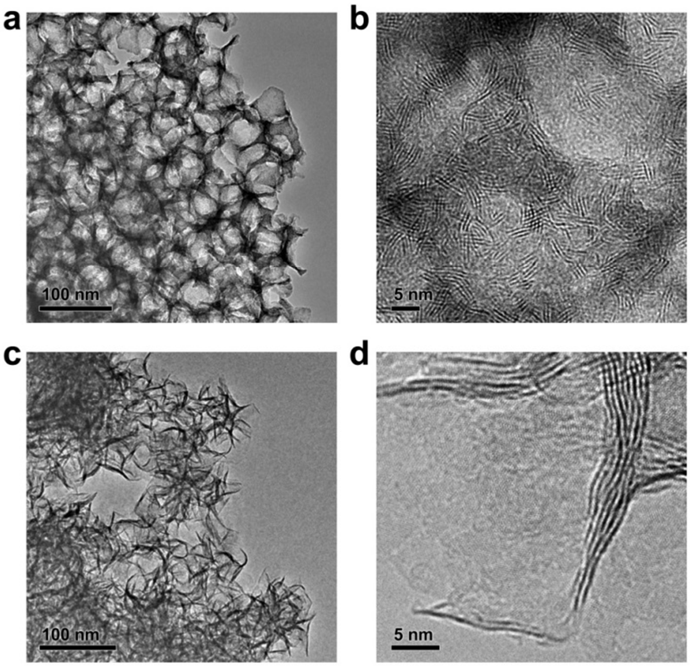

Supplementary Figure 3. TEM and HRTEM images of mPF-MoS_2 in comparison to rNS-MoS_2: (a, b) mPF-MoS_2, (c, d) rNS-MoS_2.

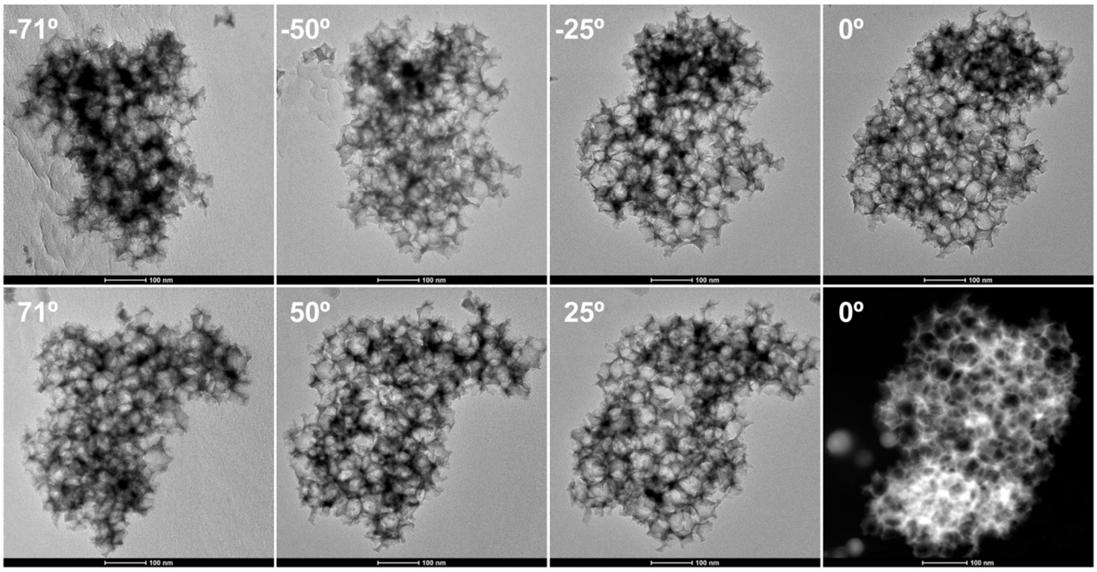

Supplementary Figure 4. TEM and HAADF-STEM images taken at different tilting angles for mPF-MoS_2. The TEM sample was prepared by dispersing the ethanol with mPF-MoS_2 on a holey carbon-covered Cu grid. The range of the tilting angles for 3D tomography is between -71 and 71 degree.

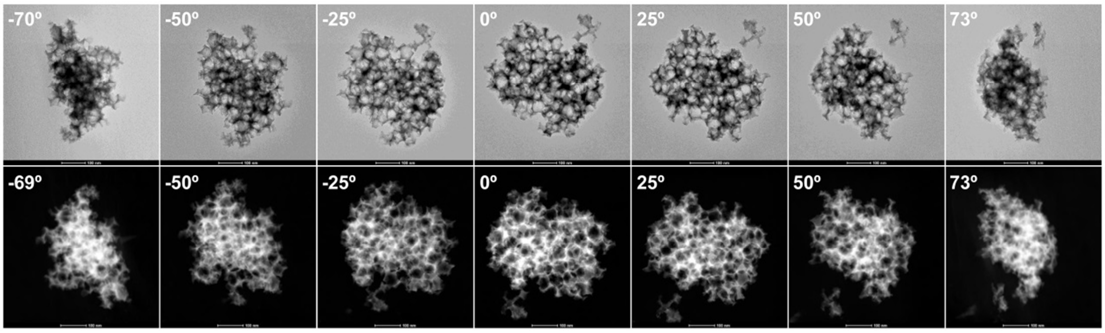

Supplementary Figure 5. TEM and HAADF-STEM images taken at different tilting angles for mPF-MoS_2. The TEM sample was prepared by dispersing the ethanol with mPF-MoS_2 on a holey carbon-covered Cu grid. The range of the tilting angles for 3D tomography is between -69 and 73 degree.

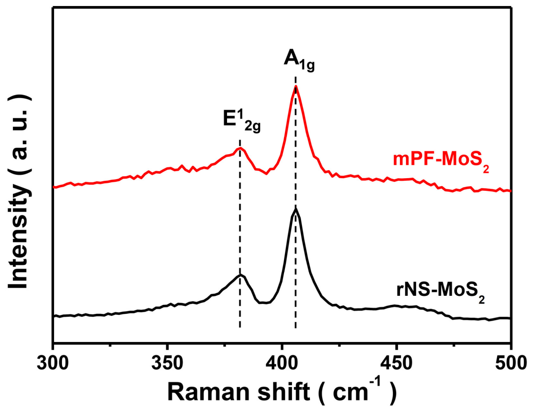

Supplementary Figure 6. Raman spectra of mPF-MoS_2 in comparison to rNS-MoS_2.

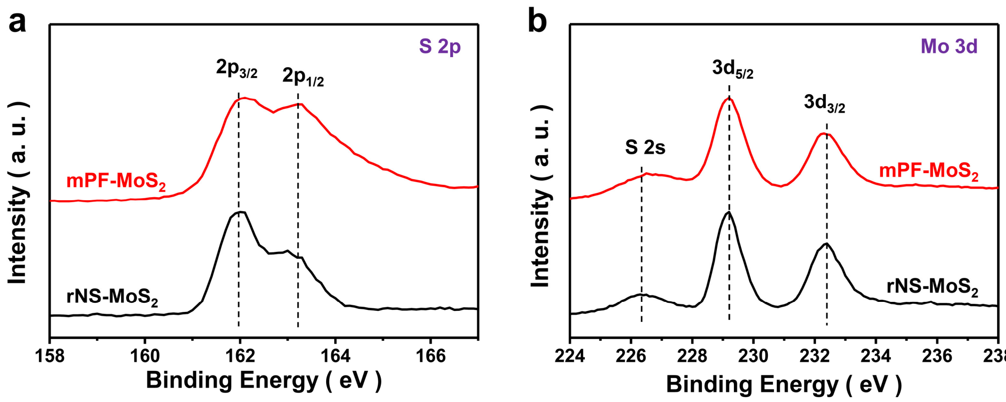

Supplementary Figure 7. XPS spectra of mPF-MoS_2 in comparison to rNS-MoS_2: (a) S 2p spectrum, (b) Mo 3d spectrum.

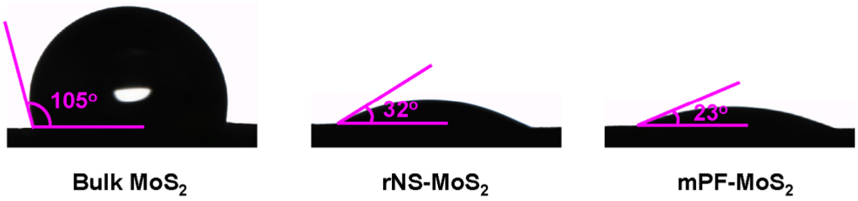

Supplementary Figure 8. Contact angle of water droplets on the surface of bulk MoS_2, rNS-MoS_2, and mPF-MoS_2, respectively.

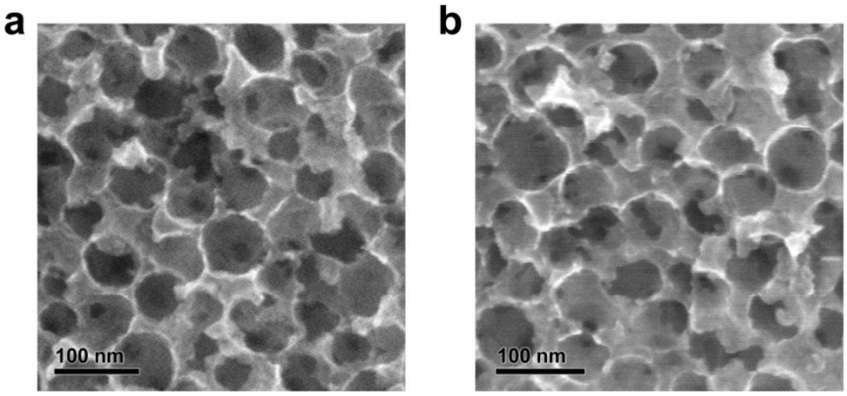

Supplementary Figure 9. (a, b) SEM images of mPF-Co-MoS_2-16.7.

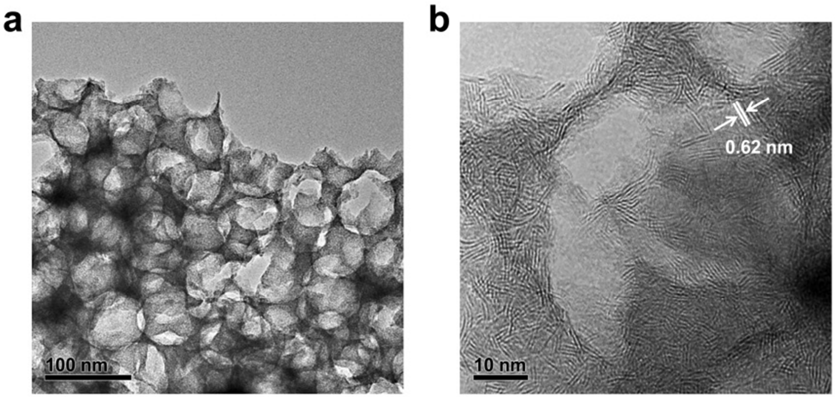

Supplementary Figure 10. (a) TEM image of mPF-Co-MoS_2-16.7. (b) HRTEM image of mPF-Co-MoS_2-16.7 with inset showing a typical MoS_2 layer distance of 0.62 nm.

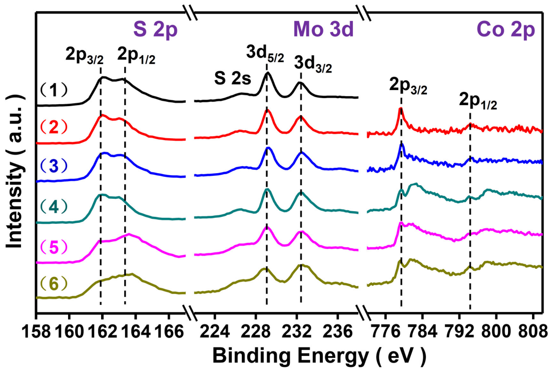

Supplementary Figure 11. S 2p, Mo 3d and Co 2p XPS spectra of a series of mPF-Co-MoS_2 samples in comparison to mPF-MoS_2. The numbers (1), (2), (3), (4), (5) and (6) represent mPF-MoS_2 and mPF-Co-MoS_2 with the Co doping contents of 3.4%, 7.6%, 16.7%, 21.1% and 31.8%, respectively.

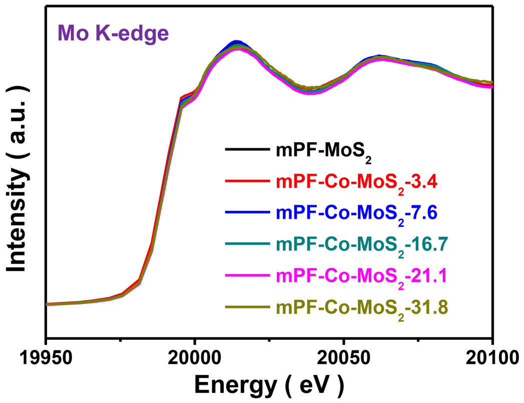

Supplementary Figure 12. Mo K-edge XANES spectra of a series of mPF-Co-MoS_2 samples in comparison to mPF-MoS_2.

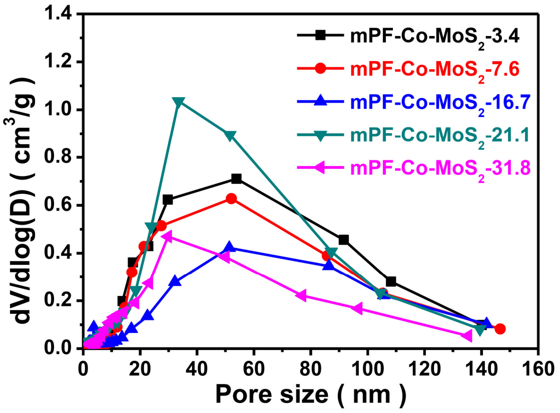

Supplementary Figure 13. Pore size distribution of mPF-Co-MoS_2 with different Co doping contents of 3.4%, 7.6%, 16.7%, 21.1% and 31.8%, respectively.

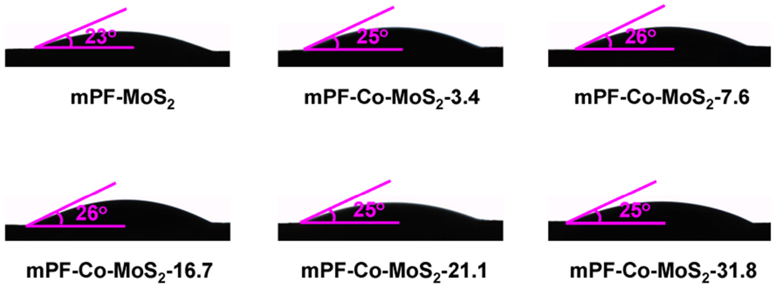

Supplementary Figure 14. Contact angle of water droplets on the surface of a series of mPF-Co-MoS_2 samples in comparison to mPF-MoS_2.

Supplementary Table 1. Summary of representative MoS_2-based HER catalysts in acidic electrolyte.

Catalyst	Catalyst amount (mg/cm^2)	Electrolyte	Overpotential at 10 mA cm^{-2}	Reference
**mPF-Co-MoS_2-16.7**	0.5	0.5 M H_2SO_4	156 mV	This work
**mPF-MoS_2**	0.5	0.5 M H_2SO_4	210 mV	This work
**H-MoS_2**	1	0.5 M H_2SO_4	167 mV	Adv. Mater., 2015, 27, 7426
**MoS_2/CNT-graphene**	0.65	0.5 M H_2SO_4	255 mV	ACS Nano, 2014, 8, 5164
**$CoMoS_3$**	0.5	0.5 M H_2SO_4	171 mV	Adv. Mater., 2016, 28, 92
**C@MoS_2 nanoboxes**	0.5	0.5 M H_2SO_4	165 mV	Angew. Chem. Int. Ed., 2015, 54, 7395
**$CoMoS_x$**	-	H_2SO_4 (PH=1)	~210 mV	Nat. Mater. 2016, 15, 197
**Double-gyroid MoS_2**	0.06	0.5 M H_2SO_4	~260 mV	Nat. Mater. 2012, 11, 963
**Strained vacancy MoS_2**	-	H_2SO_4 (PH=0.2)	170 mV	Nat. Mater. 2016, 15, 48
**MoS_2@OMC**	0.3	0.5 M H_2SO_4	182 mV	ACS Nano, 2015, 9, 3728
**MoS_2Cl-VG**	-	0.5 M H_2SO_4	160 mV	Energy Environ. Sci., 2015, 8, 862
**Exfoliated MoS_2**	-	0.5 M H_2SO_4	195 mV	J. Am. Chem. Soc., 2013, 135, 10274
**M-MoS_2**	0.043	0.5 M H_2SO_4	175 mV	Nat. Commun., 2016, 7, 10672
**Interlayer-expanded MoS_2**	0.28	0.5 M H_2SO_4	149 mV	Nat. Commun., 2015, 6, 7493
**Defect-rich MoS_2**	0.285	0.5 M H_2SO_4	195 mV	Adv. Mater., 2013, 25, 5807

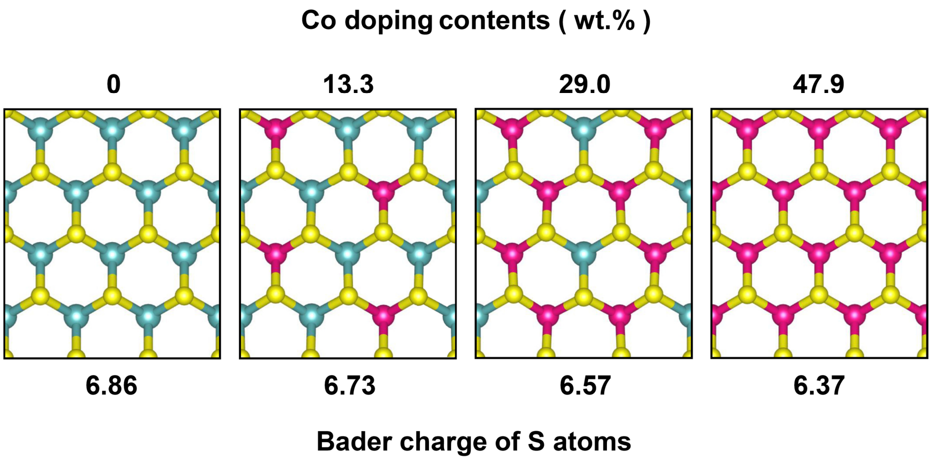

Supplementary Figure 15. Optimized structures of pure MoS_2, Co-doped MoS_2 (Co doping contents of 13.3 wt.% (Co:Mo atomic ratio of 1:2) and 29.0 wt.% (Co:Mo atomic ration of 2:1)) and CoS_2 (Co content of 47.9 wt.%), respectively, as well as their corresponding average Bader charge of S atoms. Green balls: Mo; yellow balls: S; pink balls: Co.

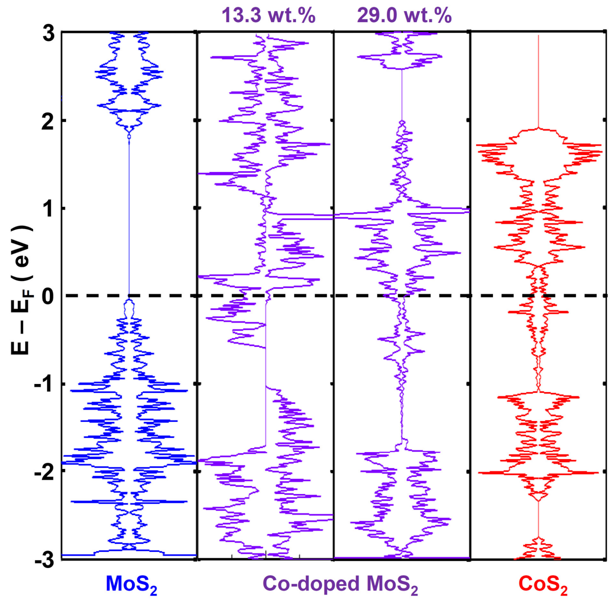

Supplementary Figure 16. Projected DOS of S atoms from pure MoS_2, Co-doped MoS_2 (Co doping contents of 13.3 wt.% (Co:Mo atomic ratio of 1:2) and 29.0 wt.% (Co:Mo atomic ration of 2:1)) and CoS_2 (Co content of 47.9 wt.%), respectively. The Co atoms are uniformly doped, and all the S atoms are in the same chemical environment.

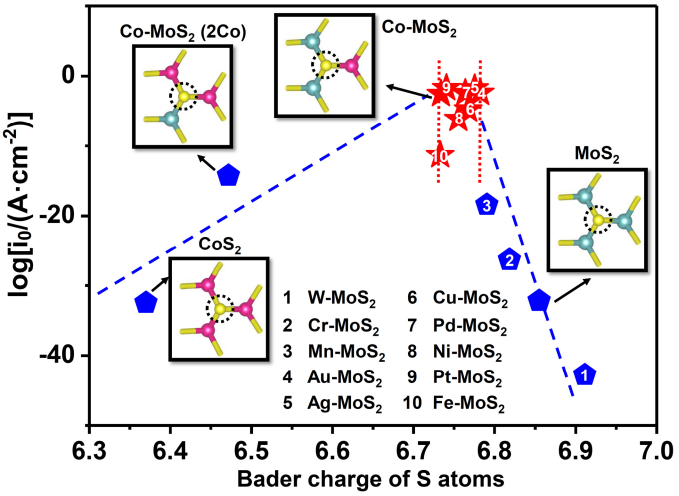

Supplementary Figure 17. Currents log(i_0) versus the Bader charge of S atoms from different doping structures of MoS_2 (i_0 represents the exchange current density), with the detailed data for each point shown in Supplementary Table 2. The S atoms with Bader charge number within the range of ~6.73 to ~6.78 own the best HER activities, which are shown as red stars. The insets are the atomic configurations of one S atom bonding with three Co, two Co and one Mo, one Co and two Mo, as well as three Mo atoms, respectively. Green balls: Mo; yellow balls: S; pink balls: Co.

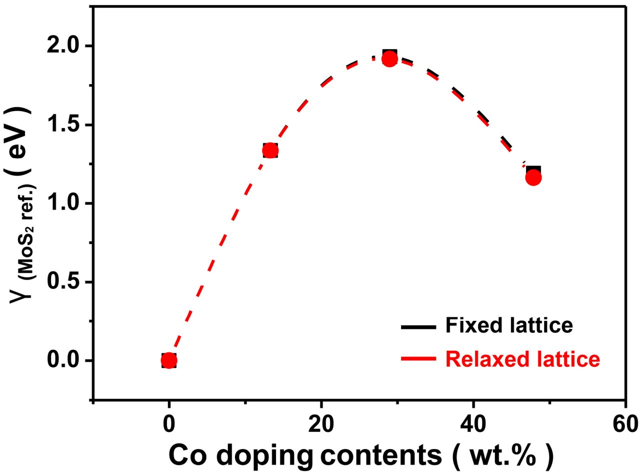

Supplementary Figure 18. Surface energy (γ) of pure MoS_2, Co-doped MoS_2 (Co doping contents of 13.3 wt.% (Co:Mo atomic ratio of 1:2) and 29.0 wt.% (Co:Mo atomic ration of 2:1)) and CoS_2 (Co content of 47.9 wt.%) per S-Mo-S formula unit (one Mo atom and two S atoms), in reference of pure MoS_2. The γ of fixed lattice and free-relaxed lattice are calculated in black and red dash lines, respectively. It can be seen that the effect of the doped Co atoms to the lattice size is almost negligible.

Supplementary Table 2. Different doping structures of MoS_2, Bader charge of S atoms neighboring to the doped metal atoms, the hydrogen adsorption free energy (ΔG_H) on S atoms and the currents $\log(i_0)$. The sequence is corresponding to the points in Figure 6d and Supplementary Figure 17, from left to right.

Structures	Bader charge of S	ΔG_H (eV)	$\log(i_0/(A \cdot cm^{-2}))$
CoS_2	6.37	-1.85	-32.46
Co-MoS_2 (2Co)[a]	6.47	-0.76	-14.26
Fe-MoS_2	6.73	0.59	-11.34
Co-MoS_2	6.73	-0.07	-2.56
Pt-MoS_2	6.74	-0.00	-1.73
Ni-MoS_2	6.76	-0.28	-6.14
Pd-MoS_2	6.76	-0.05	-2.39
Cu-MoS_2	6.77	-0.20	-4.79
Ag-MoS_2	6.78	0.01	-1.78
Au-MoS_2	6.78	-0.06	-2.44
Mn-MoS_2	6.79	1.01	-18.40
Cr-MoS_2	6.82	1.48	-26.30
MoS_2	6.86	1.83	-32.18
W-MoS_2	6.91	2.46	-42.76

[a] Two Co atoms are doped at the neighboring Mo sites. (For other metal-MoS_2 structures, one metal atom is doped at the Mo site.)

Supplementary Methods

Computational details. The hydrogen adsorption energy (ΔE_H) on S atoms is calculated by: $\Delta E_H = E(MoS_2+H) - E(MoS_2) - 1/2E(H_2)$, where $E(MoS_2+H)$ is the total energy of the metal atoms doped MoS_2 with one absorbed H atom, $E(MoS_2)$ is the total energy of the metal atoms doped MoS_2 without absorbed H atoms, and $E(H_2)$ is the energy of a gas phase hydrogen molecule. ΔG_H is calculated as: $\Delta G_H = \Delta E_H + 0.24$ eV.

The surface energy (γ) is calculated by: $\gamma = E_{tot} - [E(MoS_2) - N(Mo)*E(Mo) + N(Co)*E(Co)]$, where E_{tot} is the total energy of Co atoms doped MoS_2, $E(MoS_2)$ is the total energy of pure MoS_2, N(Mo) and N(Co) is the number of Mo or Co atoms in the unit cell, E(Mo) and E(Co) is the energy of one Mo or Co atom in reference to bulk Mo or Co metals.
